# Programmed Cell Death in the Endosperm Is a Hallmark of Seed Germination in *Viola*

**DOI:** 10.3390/ijms27073046

**Published:** 2026-03-27

**Authors:** Jacek Łuc, Monika Kwiatkowska, Aneta Słomka, Magdalena Surman, Magdalena Wilczak, Klaudia Sychta

**Affiliations:** 1 Department of Plant Cytology and Embryology, Institute of Botany, Faculty of Biology, Jagiellonian University, 9 Gronostajowa St., 30-387 Krakow, Poland; jacek.luc@student.uj.edu.pl (J.Ł.); monika.m.kwiatkowska@uj.edu.pl (M.K.); aneta.slomka@uj.edu.pl (A.S.); 2 Department of Glycoconjugate Biochemistry, Institute of Zoology and Biomedical Research, Faculty of Biology, Jagiellonian University, 9 Gronostajowa St., 30-387 Krakow, Poland; magdalena.surman@uj.edu.pl (M.S.); magdalena.wilczak@doctoral.uj.edu.pl (M.W.)

**Keywords:** plant programmed cell death, violets, Western blot, TUNEL assay, seed germination

## Abstract

Programmed cell death (PCD) is a pivotal biological process that occurs at various stages of plant development, including embryogenesis and seed germination. This study investigated whether the absence of PCD in endosperm cells is connected to the poor germination of *Viola odorata* seeds. Seeds of poorly germinating *V. odorata* and well-germinating *V.* × *wittrockiana* were either cold-stratified for 10 days or left untreated. Germination tests, tetrazolium viability tests, Western blot analyses for caspase-like proteases, and Terminal deoxynucleotidyl transferase (TdT) dUTP nick end labeling (TUNEL) assays for DNA strand break detection were performed. The results revealed that *V. odorata* seeds did not germinate, regardless of stratification or lack thereof, whereas in *V.* × *wittrockiana*, stratification significantly increased their germination capacity (34 ± 6.5% vs. 56.5 ± 9.8% in non-stratified and stratified seeds, respectively). The tetrazolium viability test revealed that *V. odorata* seeds were nonviable (100% nonviable endosperm and 96% nonviable embryos in total), whereas the seeds of *V.* × *wittrockiana* were highly viable (63% and 59% endosperm and embryos in total, respectively). Western blot analysis revealed that in the germinating seeds of *V.* × *wittrockiana*, caspase-like activity was detected in the endosperm but not in the embryos, whereas in seeds that failed to germinate, the PCD signal in the endosperm was very weak. In the seeds of *V. odorata*, caspase-like activity was detected in the embryos and endosperm collected directly after 10 days of stratification, but no signal was detected in the seeds left to germinate for one month after cold stratification. TUNEL assays revealed DNA strand breaks in the peripheral part of the endosperm in *V. odorata* and in non-germinating *V.* × *wittrockiana*, whereas in the germinating seeds of *V.* × *wittrockiana*, DNA strand breaks were detected in the endosperm cells adjacent to the embryo. These findings indicate that endosperm-localized PCD facilitates nutrient mobilization to the embryo and seems crucial for successful germination. Overall, these results suggest that PCD contributes to the regulation of seed germination in *Viola* spp.

## 1. Introduction

Programmed cell death (PCD) is observed in many stages of plant development—from fertilization to senescence—and as a response to stress factors. PCD in plants can be categorized into developmental PCD (dPCD), which contributes to the modulation of plant growth, and differentiation and environmental PCD (ePCD), which are responses to environmental stresses [1]. Environmental PCD occurs as a consequence of biotic (e.g., bacteria, viruses or fungi) or abiotic (e.g., salinity, heavy metals, drought, UV radiation, temperature or flooding) stress signals. Developmental PCD is triggered by internal factors and is involved in differentiation, e.g., xylem formation, tapetum degradation, and root cap cell death, or is initiated as a result of cell-to-cell signaling during self-incompatibility. Positional information may also influence the initiation of dPCD during the formation of aerenchyma or leaf perforation. Moreover, dPCD may be involved in plant senescence [1,2,3,4,5,6].

The mechanisms underlying dPCD, including the involvement of proteases and their inhibitors in the differentiation of tissues (e.g., tapetum, xylem, nucellus, megagametophyte, suspensor, seed coat, endosperm, and root cap) and senescence, are more comprehensively studied than those underlying ePCD [7,8,9]. A very large knowledge gap regarding the molecular basis of PCD during plant development, especially seed formation and germination, still exists [10]. Although caspases involved in animal cell death are not involved in the PCD process in plants, enzymes with caspase-like activity—referred to as caspase-like enzymes/proteases—have been identified in plants (as reviewed by [11]). The most important groups of cysteine proteases involved in developmental PCD in plants include metacaspases (MCs), vacuolar processing enzymes (VPEs), papain-like cysteine proteases (PLCPs) and cathepsin B-like proteases. MCs are active in PCD during either xylem development or lateral root cap formation [12,13]. MCs are evolutionarily conserved; they are found not only in plants but also in fungi and protists, although their activity has not been detected in animals [14]. Other proteases, namely vacuolar processing enzymes (VPEs), include those found in seeds (β-VPE) and those present in mature plants (α-VPE and γ-VPE). In the vegetative parts of mature plants, VPEs are located in lytic vacuoles. In seeds, VPEs are found in vacuoles that serve as storage compartments, where they accumulate various types of proteins. During germination, β-VPEs participate in the release of nutrients either through direct proteolysis or by activating other proteases, and therefore, vacuolar enzymes are crucial for the seed germination process [15]. Moreover, it has been observed that increased activity of VPEs leads to an acceleration of the germination process [16]. In recent years, a growing number of studies have focused on the role of papain-like cysteine proteases in plant PCD [17]. They play a key role in plant development, particularly during germination, where they participate in endosperm degradation and the delivery of nutrients to the embryo [18]. Finally, cathepsin B-like proteins are activated during seed germination, when protein mobilization occurs in the embryonic axis, and during the post-germination process, when stored proteins undergo hydrolysis in cotyledons [19].

In many species, the germination process is preceded by a period of dormancy. This may be caused by embryo immaturity or by waiting for favorable environmental conditions [20]. A common example of seed dormancy in temperate climates is the necessity of undergoing a period of cold treatment (cold stratification). This serves as an adaptation that protects plants from germination in autumn, which, due to the approaching winter, prevents the completion of the life cycle. Only after being exposed to low temperatures seeds can break the dormancy and begin to germinate in spring [21].

To achieve reproductive success, proper gametophyte development, double fertilization, embryo development, seed maturation, and subsequent germination are essential. Develompental PCD contributes significantly to all these stages [22,23]. Endosperm degradation, which results from PCD, is a key process supporting seed germination [24,25]. Endosperm PCD occurs to make room for the developing embryo and to release essential nutrients for embryo development [26]. The best-known example of PCD in the endosperm is cell death of the protein-rich aleurone layer of cereal grains [27]. In this context, PCD is manifested by the release of stored nutrients, primarily saccharides and proteins. Characteristic features also include DNA strand breaks, the presence of proteolytic enzymes, and increased levels of reactive oxygen species (ROS). Endosperm cell death in dicots is similar to that observed in cereals in morphological traits. Disruptions in endosperm PCD may lead to improper embryo development and nutrient supply, ultimately resulting in embryo death [1,28].

The plants that require cold stratification to initiate the germination process include violets (genus *Viola*, Violaceae), which are known for their decorative and medicinal properties. Many popular species, such as *Viola* × *wittrockiana* (garden pansy) and *V. odorata* (sweet violet), are cultivated as ornamental plants [29]. Violets have been widely applied in medicine and serve as sources of valuable flavonoids, saponins, and alkaloids. *V. odorata* is of particular interest and has been used for years in both traditional medicine and modern pharmacology. Plant extracts are used to treat respiratory disorders, insomnia, and headaches. Moreover, the cycloviolacin produced by this species has anticancer properties [30,31]. A significant problem for breeders and laboratory technicians is the extremely low seed germination rate under laboratory conditions, which hinders their cultivation for research purposes ([30,32] and own observations). In contrast, *V.* × *wittrockiana* is characterized by relatively high seed viability and a significantly higher germination rate [33]. Therefore, both species can serve as good subjects for verifying the hypothesis that the absence/low rate of PCD inhibits the germination of *V. odorata* seeds, whereas the high activity of this process in the endosperm of *V.* × *wittrockiana* contributes to the high germination rate of the seeds of this species.

## 2. Results

### 2.1. The Seed Germination Frequency Was Significantly Higher in V. × wittrockiana than in V. odorata

The seeds of *V.* × *wittrockiana* exhibited a relatively high germination frequency after one month: 34% of the seeds without cold stratification (NS) and 56.5% of the seeds after 10 days of cold stratification (CS) germinated. In contrast, the seeds of *V. odorata* did not germinate either upon cold stratification or without CS (0% germination frequency) (Table 1).

### 2.2. The Seed Viability Was Significantly Greater in V. × wittrockiana than in V. odorata

**Figure 1:**
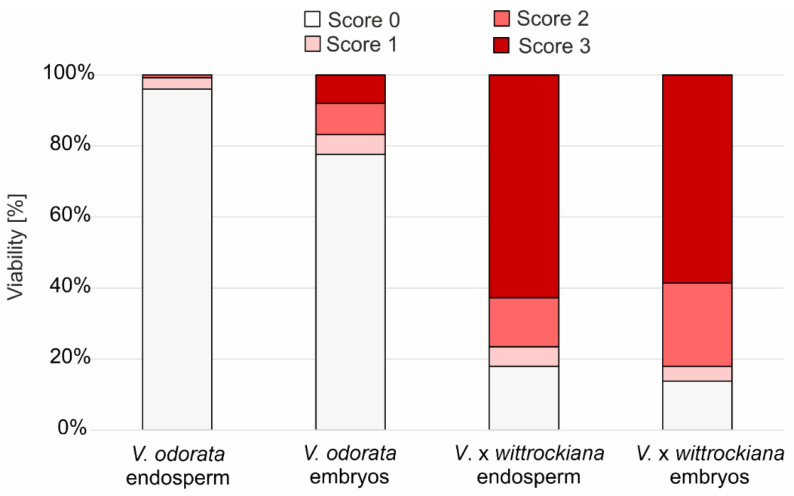
Results of tetrazolium viability test for embryos and endosperm of *V. odorata* and *V.* × *wittrockiana* after 10 days of cold stratification, scored on a four-point staining scale (0–3). Score 0—no visible staining, embryo/endosperm nonviable; score 1—<50% of embryo/endosperm surface stained red; score 2—>50% of embryo/endosperm surface stained red; score 3—100% embryo/endosperm surface stained red.

**Figure 2:**
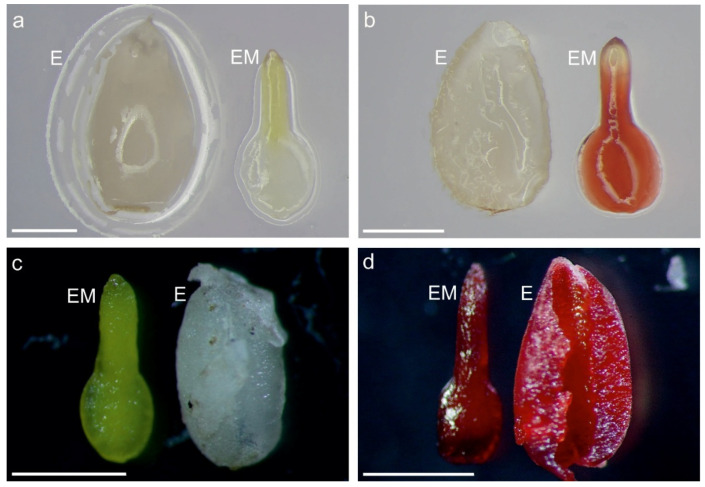
Representative stereomicroscopic images of tetrazolium-stained seeds (embryos and endosperm) of *V. odorata* with staining score 0 for both embryo and endosperm (**a**) as well as with score 0 for endosperm and score 2 for the embryo (**b**) and of *V.* × *wittrockiana* with score 0 for both embryo and endosperm (**c**) and with score 3 for both embryo and endosperm (**d**). EM—embryo, E—endosperm. Bars = 1 mm.

No *V. odorata* seeds received a score of 3 for embryo or endosperm viability simultane (Figure 1). On the basis of their stainability, 78.6% of *V. odorata* embryos and 97% of their endosperms were rated as 0, according to the accepted criteria, indicating that all *V. odorata* seeds were nonviable. In the case of *V.* × *wittrockiana*, 51% of the seeds had both endosperm and embryos with a staining rating of 3, which means that more than half of the seeds sown were completely viable. On the other hand, 13.1% of *V.* × *wittrockiana* seeds had a staining rating of 0 for both the embryo and endosperm, which means that only a minority of the seeds were completely nonviable (Figure 1, Table S1). The remaining 35.9% of the seeds presented various combinations of endosperm and embryo stainability between classes 0 and 3 (Figure 2, Table S1). In total, 8% (at a stainability level of 3) of the *V. odorata* embryos were viable, and 58.6% of *V.* × *wittrockiana* were viable. In contrast, the endosperm was viable in 0% of the former species and 62.8% of the latter (Figure 1, Table S1).

The differences between the two species were statistically significant based on the chi-square test (*p* < 0.0001). Cramér’s V was slightly greater for the endosperm than for the embryos (0.79 vs. 0.67), suggesting that the endosperm had only a slightly stronger impact on overall seed viability.

### 2.3. Programmed Cell Death Involving Caspase-like Proteases Is a Hallmark of Germinating Seeds

**Figure 3:**
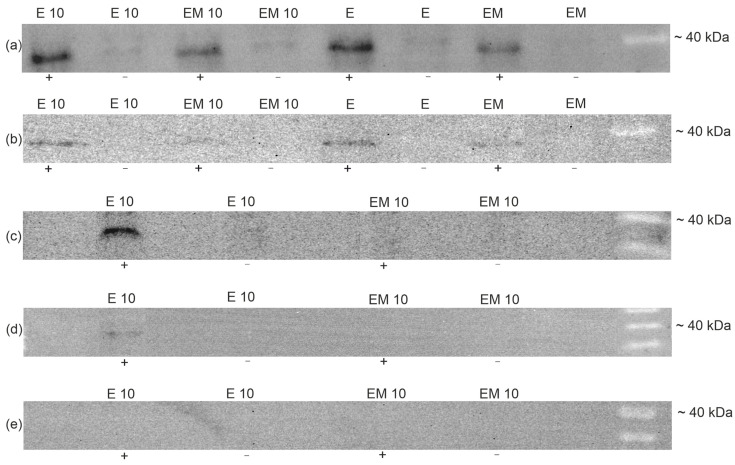
Western blot analysis of expression of caspase-like proteases in embryos (EM) and endosperms (E) of seeds after 10 days of cold stratification (E 10; EM 10) and without cold stratification (E; EM) of *V. odorata* (**a**) and *V.* × *wittrockiana* (**b**), collected immediately after treatment, and for embryos and endosperm of germinating ((**c**), collected at the moment germination began, when the radicle was visible) and non-germinating ((**d**), collected one month after sowing) seeds of *V.* × *wittrockiana* and non-germinating (collected one month after sowing) seeds of *V. odorata* (**e**). ‘+’ and ‘−’ indicate the presence or absence of biotin-xVAD-fmk in the samples.

The expression of caspase-like enzymes was detectable in both the embryos and endosperm of both the NS and CS seeds of *V. odorata* (Figure 3a) and *V.* × *wittrockiana* (Figure 3b). In the seeds of *V.* × *wittrockiana* collected during germination after 10 days of cold treatment, a strong signal of caspase-like proteases was detected only in the endosperm (Figure 3c). In stratified seeds of *V.* × *wittrockiana* that failed to germinate within one month after sowing, there was a weak detectable signal from caspase-like proteases in the endosperm (Figure 3d). In stratified, 1-month non-germinating seeds of *V. odorata*, signals from caspase-like proteases were not detected in either the endosperm or the embryos (Figure 3e).

### 2.4. DNA Strand Breaks Were Visible in the Endosperm of the Seeds Capable of Germination

**Figure 4:**
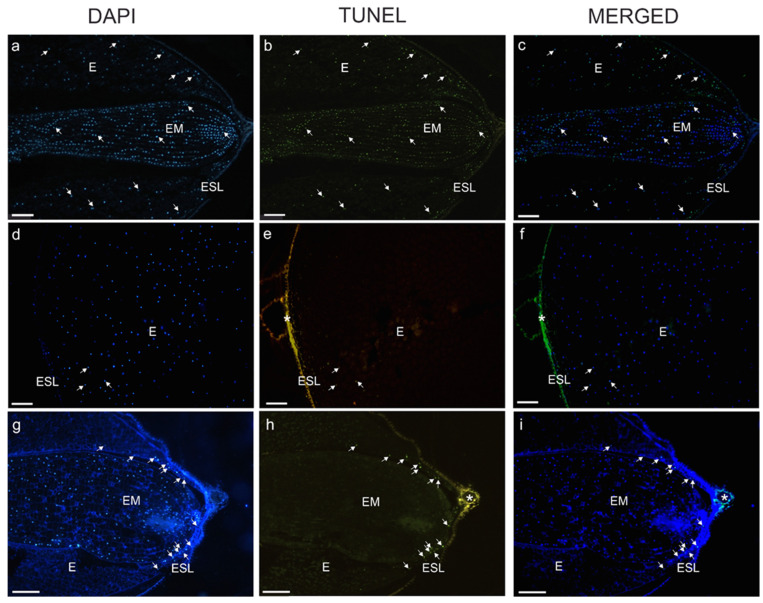
Longitudinal sections of *V. odorata* seeds after 10 days of cold stratification, collected directly after treatment (**a**–**f**) and non-germinating seeds (**g**–**i**), stained with DAPI dye and TUNEL assay (**c**,**f**,**i**—merged DAPI dye and TUNEL assay). Endosperm and embryos (**a**–**c**,**g**–**i**), endosperm (**d**–**f**). E—endosperm, EM—embryo, ESL—the layer under the seed coat (tegmen). Bars = 100 µm. The arrows indicate TUNEL-positive nuclei and the asterisks indicate the autofluorescent regions of the seed coat.

A TUNEL assay performed on the seeds of *V. odorata* CS revealed DNA strand breaks in the nuclear DNA of the cells of both the embryos and the endosperm (Figure 4a–c and Figure S1a,b). However, TUNEL-positive nuclei were mostly present in the peripheral parts of the endosperm (Figure 4d–f and Figure S1c,d). Only a few *V. odorata* NG seeds had TUNEL-positive nuclei in the peripheral endosperm (Figure 4g–i and Figure S1e,f). Furthermore, most endosperm nuclei were not stained with DAPI (Figure 4g).

**Figure 5:**
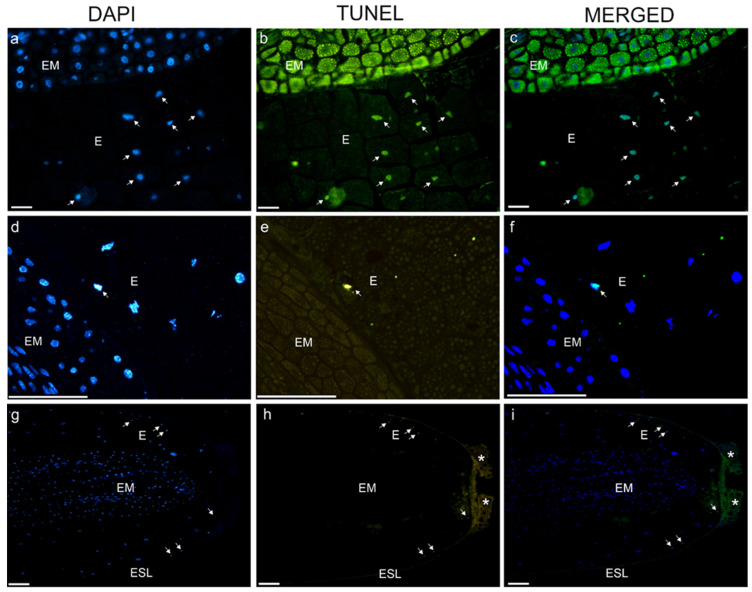
Longitudinal sections of *V.* × *wittrockiana* seeds after 10 days of cold stratification, collected directly after treatment (**a**–**c**), germinating seeds (**d**–**f**) and non-germinating seeds (**g**–**i**), stained with DAPI dye and TUNEL assay (**c**,**f**,**i**—merged DAPI dye and TUNEL assay). E—endosperm, EM—embryo, ESL—the layer under the seed coat (tegmen). Bars = 25 µm (**a**–**c**) and 100 µm (**d**–**i**). The arrows indicate TUNEL-positive nuclei and the asterisks indicate the autofluorescent regions of the seed coat.

In *V.* × *wittrockiana* CS seeds, TUNEL-positive nuclei were detected mostly in the endosperm (Figure 5a–c and Figure S2a,b). Moreover, in the germinating seeds (G) of *V.* × *wittrockiana*, TUNEL-positive nuclei were detectable only in the layer adjacent to the embryo (Figure 5d–f and Figure S2c,d). In the NG seeds of *V.* × *wittrockiana*, similar to those in *V. odorata*, TUNEL-positive nuclei were mostly present at peripheral parts of the endosperm (Figure 5g–i and Figure S2e,f).

## 3. Discussion

Programmed cell death is a key process that occurs at various stages of plant development. These include the first stages of ontogeny—embryogenesis—and then seed germination. Species of the genus *Viola* are exceptionally good models for studying PCD in seeds because they have albuminous seeds, and the reserves stored in the endosperm provide a source of nutrients for the germinating seedling, unlike the model plant *Arabidopsis thaliana*, which has exalbuminous seeds. However, the role of PCD in seed maturation and germination has not been well understood until recently [34]. The results obtained in this study confirm the role of PCD in the induction of germination. Western blot analysis revealed that caspase-like activity occurred in the endosperm and embryos of seeds either without stratification or following a period of cold treatment in species with high germination capacity (*V.* × *wittrockiana*) as well as in a species that failed to germinate (*V. odorata*) after cold treatment (Figure 3a,b). These results correspond to those of the TUNEL assay, in which degenerating nuclei were observed in the embryo and endosperm of seeds collected immediately after stratification (Figure 4a–c) for *V. odorata* and in the endosperm of *V.* × *wittrockiana* (Figure 5a–c). Considering that in *V.* × *wittrockiana*, the percentage of germinating seeds was 34% and 56.5% for non-stratified and stratified seeds, respectively (Table 1), the PCD hallmarks observed in the embryos may have resulted from the random selection of seeds for Western blot analysis, including both those capable and incapable of germination, in which the embryo is undergoing degeneration. PCD in the embryo may represent a normal aspect of development or may be a pathological symptom. During embryogenesis, PCD is involved in the differentiation and development of the provascular tissue [1,35]. Once the embryo reaches maturity, PCD leads to the formation of pits and vessels [36]. Extensive evidence indicates that the embryo plays an active role in germination by releasing signals that initiate reserve degradation and promote the loosening of the endosperm [37]. Activation of PCD in the embryo may also be a pathological symptom induced by increased levels of ROS in the cells or by disturbances in zinc homeostasis [38,39]. In the case of *V.* × *wittrockiana* seeds that initiated the germination process, caspase-like activity was observed only in the endosperm but was undetectable in the embryo (Figure 3c). On the other hand, caspase-like activity was detected in the embryos of *V. odorata* and *V.* × *wittrockiana* seeds collected directly after stratification and those with no stratification (Figure 3a,b). Therefore, caspase-like activity may be associated with developmental defects in the seed and impaired germination capacity. In *V.* × *wittrockiana* seeds that are not capable of germinating, the intensity of caspase-like activity in the endosperm is significantly lower than that in germinating seeds (Figure 3c,d). These results suggest that the increased caspase-like activity inducing the PCD process in the endosperm is crucial for the initiation of germination, whereas weak caspase-like activity in the endosperm may contribute to the inhibition of the germination process. In *V. odorata*, in seeds left for one month to germinate, PCD hallmarks were only weakly detected by the TUNEL assay in both the endosperm and the embryo and were absent in the Western blot analysis. This suggests that all tissues within the seed had undergone cell death during this time without germination (Figure 3e and Figure 4g–i).

These findings correspond with the results of the TTC test, where both the endosperm and the embryos were found to be nonviable (Figure 1 and Figure 2, Table S1). The TTC test is based on respiration activity, allowing the assessment of cell viability and metabolic activity. TTC test revealed that the seeds of *V.* × *wittrockiana* were predominantly viable, suggesting that no developmental abnormalities occurred in either the endosperm or the embryos (Figure 1 and Figure 2, Table S1), which is reflected in their retained germination capacity. TUNEL-positive nuclei were observed at the periphery of the endosperm in *V. odorata* seeds, whereas in the germinating seeds of *V.* × *wittrockiana*, PCD occurred in the cells close to the embryo (Figure 4a–f and Figure 5d–f). The spatial separation of the endosperm degradation process can be a deliberate process, i.e., when the endosperm located at the embryo undergoes degradation, allowing it to be nourished. On the other hand, this spatial distribution of degrading cells may reflect the effect of time, as non-germinating seeds were analyzed only one month after stratification, when the degradation process could be observed only at its final stage. Shinozaki et al. [40] demonstrated that plant autophagy maintains seed germination ability. Autophagic flux was sustained in endosperm cells during storage, and a defect in autophagy led to the accumulation of oxidized proteins and accelerated endosperm cell death. Autophagy preserves endosperm quality during seed storage by limiting aging-dependent oxidative damage and cell death, thereby enabling the endosperm to perform its optimal functions during seed germination. In *V. odorata* seeds, such a defect may be possible, as indicated by the lack of endosperm viability one month after sowing. Considering that during embryo invasive growth, the surrounding region of the endosperm is eliminated to ensure proper development [25], the first hypothesis put forward at the end of the Introduction section seems plausible.

In summary, the results of this study indicate the involvement of programmed cell death in the endosperm during the seed germination process. However, further research is needed to elucidate the mechanisms underlying this process during germination and to identify the factors that trigger PCD. Further comprehensive studies on the relationship between PCD and germination capacity will contribute to a better understanding of the role of PCD in the reproductive success of plants.

## 4. Materials and Methods

### 4.1. Pretreatment of Seeds

Seeds of *V. odorata* and *V.* × *wittrockiana* were obtained from a commercial seed supplier (Legutko, Rawicz, Poland). The seeds of both species were divided into two groups: those pretreated for 10 days by cold at 4 °C, wet stratification (CS) and those with no stratification (NS). The seeds were sterilized in 70% ethanol for 90 s, followed by 50% commercial bleach for 15 min and rinsed thrice with sterile distilled water at 3, 5 and 10 min, respectively. Afterward, they were sown on Petri dishes lined with sterilized filter paper soaked in sterile deionized water and sealed with parafilm.

Seeds of CS were kept in the growth room under stable conditions at 25 ± 3 °C with a 16 h photoperiod under cool-white fluorescent lamps (flux 70–100 μmol m^−2^s^−1^) until germination (germinating seeds—G). To collect the imbibed seeds that failed to germinate (NG), a one-month waiting period was used to ensure that they lacked germination capacity.

### 4.2. Determination of Seed Germination Frequency

The germination frequency of NS and CS seeds was measured one month after sowing. For both species, five independent sets of 40 seeds each were analyzed. In total, 200 seeds per group were counted.

### 4.3. Tetrazolium (TTC) Viability Test

The seed coats of the CS seeds of *V.* × *wittrockiana* (n = 145) and *V. odorata* (n = 125) were gently removed using a scalpel under a stereomicroscope. Dehulled seeds were incubated overnight in 1% triphenyltetrazolium chloride (TTC) (Sigma-Aldrich, Saint Louis, MO, USA) at room temperature. Tetrazoline salt stains the viable cells red because of respiratory processes occurring in living cells [41,42]. The samples were subsequently transferred to glycerol to stop the staining reaction. In the next step, the embryos and endosperm were delicately separated and then analyzed using a stereomicroscope equipped with a camera (Opta-tech, Hdmi Cam, Warsaw, Poland). Viable cells were stained red, and nonviable cells remained unstained. To assess seed viability, a scale from 0 to 3 was established: 0—colorless; 1—less than 50% of the surface of the embryo/endosperm color change; 2—more than 50% of the surface of the embryo/endosperm color change; and 3—whole embryo/endosperm surface stained red, according to Siuta et al. [43], with some modifications. Only the seeds scoring 3 on the viability scale for both the embryo and endosperm were considered fully viable.

### 4.4. Protein Extraction and Biotinylated Inhibitor Blot Analysis for the Presence of Caspase-like Proteases

Embryos or endosperm from stratified (CS), non-stratified (NS), germinating (G) or non-germinating (NG) seeds (collected from up to 50 seeds) of *V. odorata* and *V.* × *wittrockiana* were homogenized with extraction buffer (50 mM Na-acetate, 50 mM NaCl, 1 mM EDTA, diluted in distilled water; pH 5.5 adjusted with HCl) using a Retsch MM 400 (Haan, Germany) bead mill (30 Hz for 2 min) and then centrifuged (1200 rpm for 3 min). The supernatants were transferred to new Eppendorf tubes. The protein concentration was estimated using Bradford reagent (Sigma-Aldrich, Saint Louis, MO, USA) and bovine serum albumin (BSA) as a standard [44]. Absorbances for the calibration curve (0, 0.05, 0.1, 0.2, 0.3, 0.4, and 0.5 µg/µL) were measured, and the samples were analyzed at 595 nm using a microplate reader (Tecan Infinite 200 PRO, Männedorf, Switzerland). The protein concentration in each sample was then adjusted to 0.5 µg/µL using deionized water. Each extract was frozen and kept at −20 °C for further analysis.

To identify caspase-like proteases, 10 µL of each protein extract (i.e., 5 µg of protein) was mixed with 1.2 µL of 0.4 M DTT and 1.2 µL of biotin-xVAD-fmk (Caspase I inhibitor, Biotin-conjugate, Calbiochem, MW 672.8 g/L) and incubated for 30 min at room temperature. A protein solution without a biotin-xVAD-fmk inhibitor was used as a control. Both the biotin-xVAD-fmk-conjugated proteins and the control protein were subjected to SDS-PAGE and transferred to polyvinylidene difluoride (PVDF) membranes (Sigma-Aldrich, Saint Louis, MO, USA) as described by Sychta et al. [17]. The proteins on the membrane were blocked with 2% BSA and then incubated for 30 min with a 1:25,000 dilution of biotin-binding streptavidin–horseradish peroxidase conjugate (Thermo Fisher Scientific, Waltham, MA, USA) diluted 25,000-fold for 30 min. Detection was performed with an enhanced chemiluminescence kit with West Femto Maximum Sensitivity Substrate (Thermo Fisher Scientific, Waltham, MA, USA) using a detection system with a CCD camera (GeneGenome5 chemiluminescence system, Syngene, Cambridge, UK) in ECL mode. Chemi ECL mode was applied for approximately 3 min, depending on the intensity of the protein bands. The visible band indicated the presence of caspase-like proteases in the protein extract. Each membrane was prepared in triplicate.

### 4.5. TUNEL Assay

To visualize DNA strand breaks, a Terminal deoxynucleotidyl transferase (TdT) dUTP nick end labeling (TUNEL) assay was performed according to the methods of Tripathi et al. [45] and Uzelac et al. [46], with modifications. Stratified (CS), non-stratified (NS), germinating (G) or non-germinating (NG) seeds (10 seeds for each group) of *V. odorata* and *V.* × *wittrockiana* were fixed in 4% formaldehyde (FA) freshly prepared from paraformaldehyde (PFA) and 2% glutaraldehyde (GA) solution in phosphate-buffered saline (PBS) (pH 7.2). During the first hour, the material was kept in a vacuum desiccator for better infiltration of the fixative solution and then kept at 4 °C overnight. The seeds were subsequently rinsed with PBS and dehydrated in an ethanol dilution series (30%, 50%, 70%, 90% and 100%; two times for 30 min for each dilution). Dehydrated samples were incubated with ice-cold acetone twice for 30 min. Then, the seed samples were infiltrated gradually on ice with Technovit 8100 (Kulzer, Hanau, Germany) mixed with acetone at 1:3, 1:1, and 3:1 ratios, each for 1 h. Afterward, the samples were infiltrated with pure Technovit 8100 overnight at 4 °C, embedded in Technovit 8100 Hardener (made according to the manufacturer’s protocol) and stored at 4 °C for polymerization. Embedded samples were sectioned to 5 µm using a rotary microtome HM 340 E (Epredia, Kalamazoo, MI, USA), transferred to Polysine™-coated slides (Thermo Fisher Scientific, Waltham, MA, USA), dried and kept at 4 °C until future use. Prior to the TUNEL assay, the slides were incubated for 15 min on ice with permeabilization solution (1.2% sodium citrate with 10% BSA) and rinsed with PBS. The sections were transferred to a humid chamber, incubated with proteinase K working solution (20 µg/mL in Tris-HCl, pH 7.4; Sigma-Aldrich, Saint Louis, MO, USA) for 30 min at 37 °C and then rinsed with PBS. The samples were subsequently subjected to TUNEL using an in situ cell death detection kit (Roche, Penzberg, Germany), according to the manufacturer’s protocol. Simultaneously, positive ((pretreated with DNase I; Roche, Penzberg, Germany) at 37 °C for 20 min before incubation with TdT-mediated X-dUTP nick end labeling)) and negative (with TdT enzyme but without incubation with TdT-mediated X-dUTP nick end labeling) controls were prepared (Figure S3). Altogether, positive controls, as well as investigated samples, were incubated with TdT-mediated X-dUTP nick end labeling in the dark for 1 h at 37 °C. After incubation, the sections were rinsed with PBS and stained with 0.1 µg/mL DAPI (4′,6-diamidino-2-phenylindole) dissolved in PBS (Sigma-Aldrich, Saint Louis, MO, USA) for 5 min in the dark to stain the nuclei. The samples were examined using a fluorescence microscope (DM6 B; Leica, Düsseldorf, Germany). All experiments were performed in triplicate and repeated thrice.

### 4.6. Statistical Analyses

An independent Student’s *t*-test was performed to determine significant differences between the germination rate of *V.* × *wittrockiana* seeds with and without cold stratification. Differences in embryo and endosperm viability between species were tested using the chi-square (χ^2^) test of independence, followed by Cramér’s V test to estimate the impact of each tissue on seed viability.

## 5. Conclusions

(1) The seed germination frequency and seed viability were significantly greater in *V.* × *wittrockiana* than in *V. odorata*.
(2) Programmed cell death in the endosperm induced by caspase-like proteases is a process that accompanies seed germination.
(3) DNA strand breaks were observed in the endosperm of seeds capable of germinating.

## Figures and Tables

**Table 1:** Mean germination frequency (after one month since sowing) of non-treated *V.* × *wittrockiana* and *V. odorata* seeds or seeds subjected to 10 days of cold stratification. Letters (a, b, c) indicate statistically significant differences based on independent Student’s *t*-test (*p* < 0.05).

Species	Germination Frequency Without Prior Cold Stratification [% ± SD]	Germination Frequency After 10 Days of Cold Stratification [% ± SD]
*V. odorata*	0 ± 0 ^a^	0 ± 0 ^a^
*V.* × *wittrockiana*	34.0 ± 6.5 ^b^	56.5 ± 9.8 ^c^

## Data Availability

The original contributions presented in this study are included in the article/Supplementary Materials. Further inquiries can be directed to the corresponding authors.

## Supplementary Materials

The following supporting information can be downloaded at: https://www.mdpi.com/article/10.3390/ijms27073046/s1.
