# Supplementary material for: Programmed Cell Death in the Endosperm Is a Hallmark of Seed Germination in Viola
Source: Int J Mol Sci. 2026 Mar 27;27(7):3046. doi: 10.3390/ijms27073046 (PMC13072983; doi:10.3390/ijms27073046)
Supplement: Supplementary file 1 [file ijms-27-03046-s001.zip › ijms-4174502-supplementary.pdf]

Table S1. Embryos and endosperm frequency in 0-3 classes of stainability in tetrazolium viability test of *V. odorata* (n = 125) and *V.* x *wittrockiana* (n = 145) seeds.

V. odorata			*V.* x *wittrockiana*		
Arbitrary established classes of stainability		Number and frequency [%]	Arbitrary established classes of stainability		Number and frequency [%]
endosperm	embryo		endosperm	embryo	
0	0	96 [77.8]	0	0	19 [13.1]
	1	7 [5.6]		1	1 [0.7]
	2	11 [8.8]		2	6 [4.1]
	3	6 [4.8]		3	0 [0]
1	0	1 [0.8]	1	0	0 [0]
	1	0 [0]		1	3 [2.1]
	2	0 [0]		2	2 [1.4]
	3	3 [2.4]		3	3 [2.1]
2	0	0 [0]	2	0	1 [0.7]
	1	0 [0]		1	1 [0.7]
	2	0 [0]		2	10 [6.9]
	3	1 [0.8]		3	8 [5.5]
3	0	0 [0]	3	0	0 [0]
	1	0 [0]		1	1 [0.7]
	2	0 [0]		2	16 [11]
	3	0 [0]		3	74 [51]

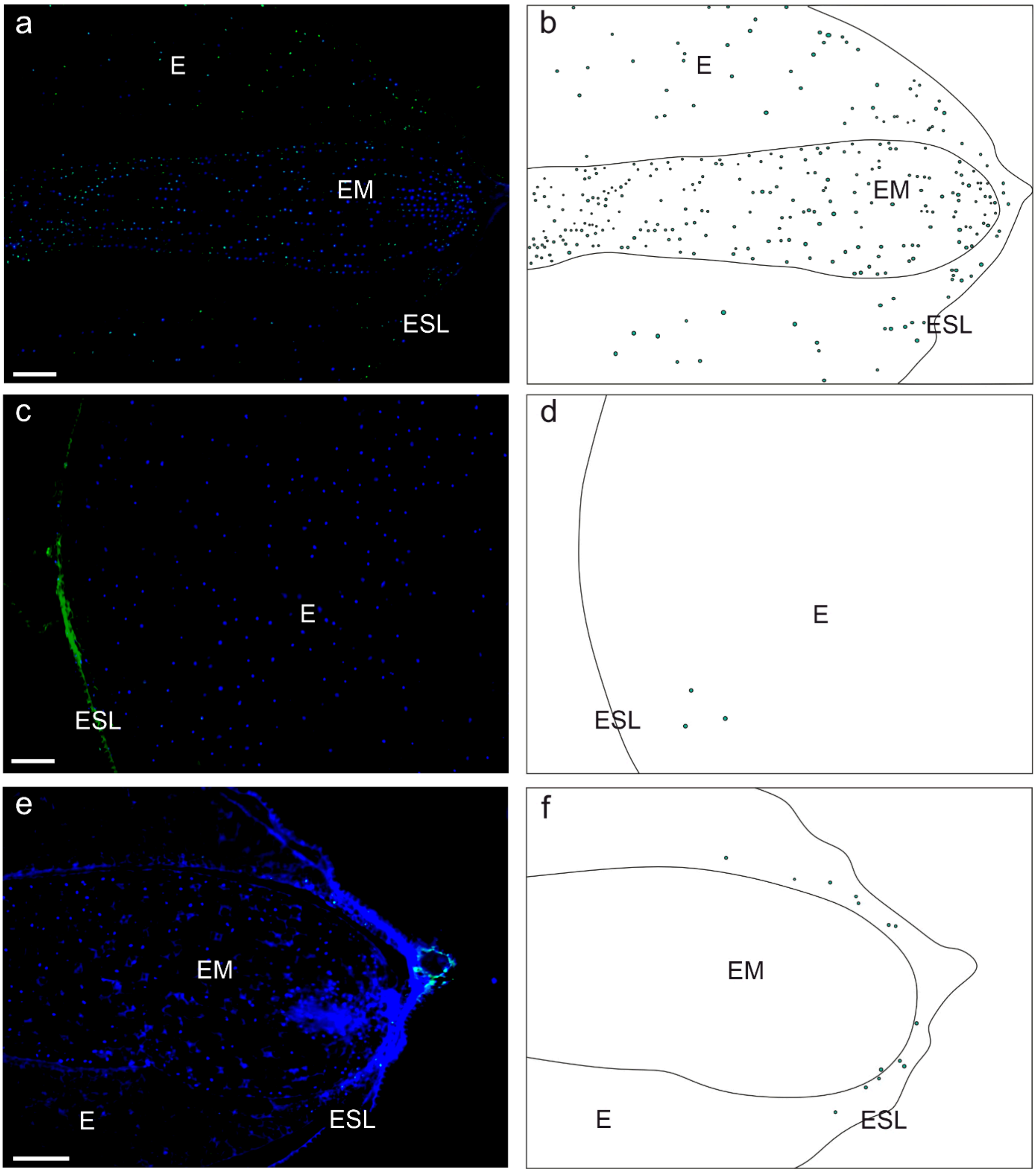

Figure S1. Longitudinal sections (a, c, e) and the corresponding schematic drawings (b, d, f) of *V. odorata* seeds after 10 days of cold stratification collected directly after treatment (a, b), and non-germinating seeds (c-f) stained with DAPI dye and TUNEL assay (MERGED). The marked points on the schemat-ics indicate the TUNEL-positive nuclei. E – endosperm, EM – embryo, ESL – the layer under the seed coat (tegmen). Bars = 100 μm.

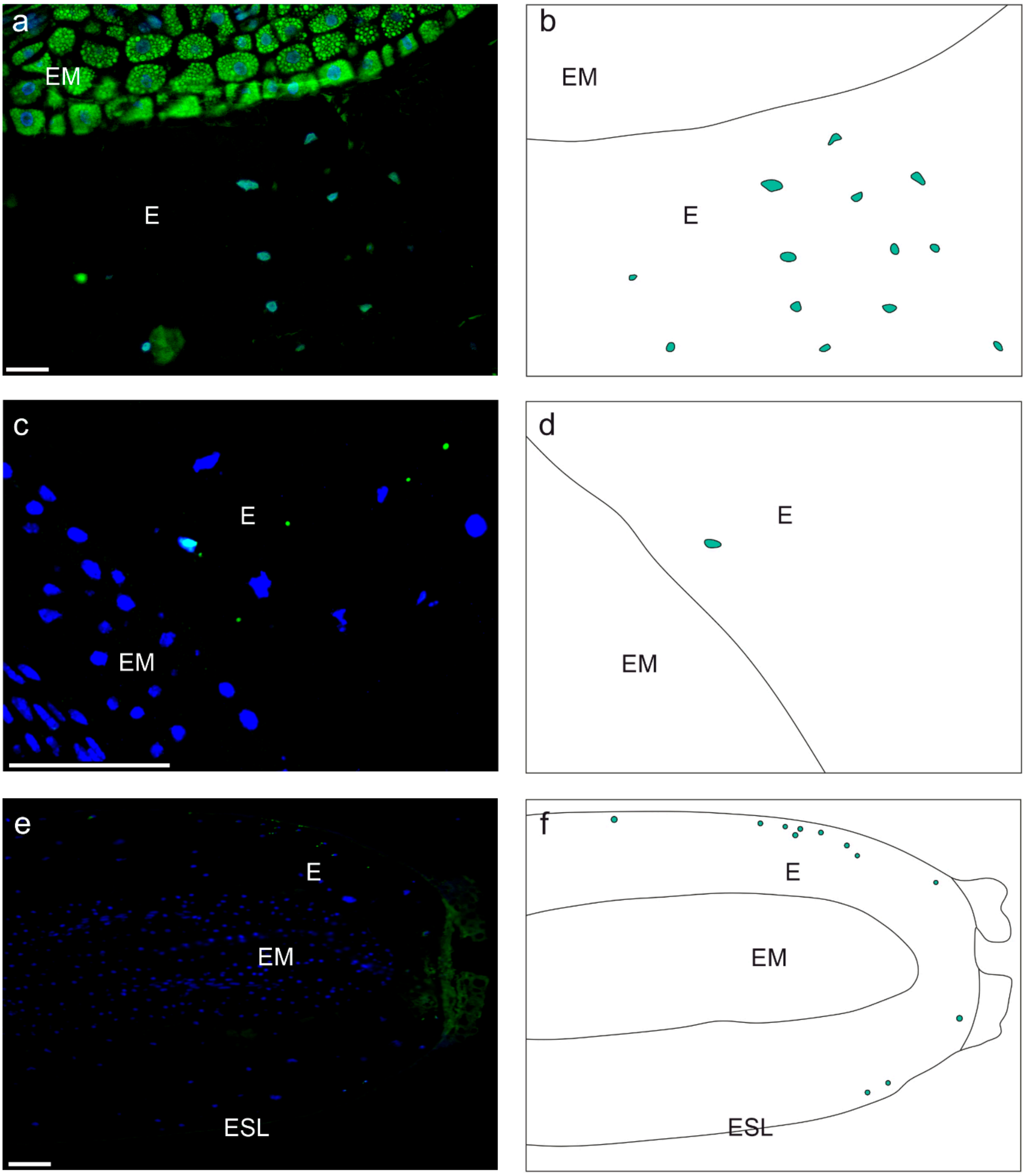

Figure S2. Longitudinal sections (a, c, e) and the corresponding schematic drawings (b, d, f) of *V.* × *wittrockiana* seeds after 10 days of cold stratification collected directly after treatment (a, b), germinating seeds (c, d) and of non-germinating seeds (e, f) stained with DAPI dye and TUNEL assay (MERGED). The marked points on the schematics indicate the TUNEL-positive nuclei. E – endosperm, EM – embryo, ESL – the layer under the seed coat (tegmen). Bars = 25 µm (a-b) and 100 µm (c-f).

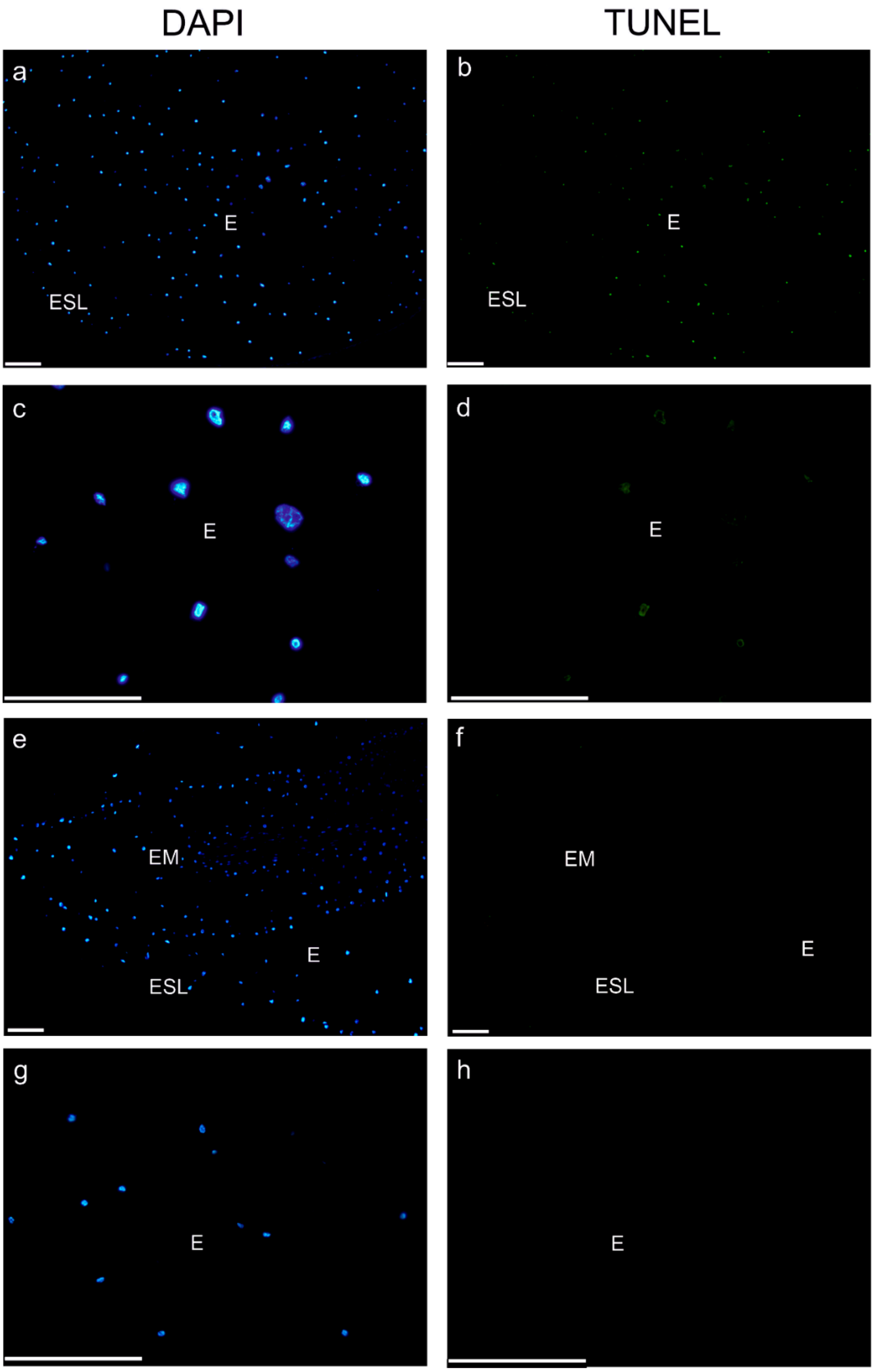

Figure S3. Longitudinal sections of the seeds after 10 days of cold stratification collected directly after treatment of *V.* x *wittrockiana* treated with DNase – positive control (a-d), and seeds of *V. odorata* treated without TdT enzyme solution – negative control (e-f) stained with DAPI and TUNEL assay. E – endosperm, EM – embryo, ESL – the layer under the seed coat (tegmen). Bars = 100 μm in (a-h).
